# Research progress of SOCS in autoimmune diseases: mechanisms and therapeutic implications

**DOI:** 10.3389/fimmu.2025.1604271

**Published:** 2025-07-18

**Authors:** Rongqi Cui, Jing Zhang

**Affiliations:** ^1^ China Medical University, Shenyang, Liaoning, China; ^2^ Department of Rheumatology and Immunology, Shengjing Hospital of China Medical University, Shenyang, Liaoning, China

**Keywords:** SOCS, autoimmune diseases, immunity, JAK-STAT, cytokines

## Abstract

Suppressor of cytokine signaling (SOCS) is a family of regulatory factors whose expression can be induced by various cytokines, growth factors, and hormones. Autoimmune diseases, chronic inflammatory conditions caused by abnormal immune responses, involve overactive T-cells and B-cells, excessive autoantibody production, and damage to multiple organs and systems. The pathogenic mechanisms of autoimmune diseases are complex, and SOCS proteins, particularly SOCS1, SOCS2, SOCS3, and SOCS5, regulate cytokine receptor signaling through distinct mechanisms, thereby participating in the development and progression of autoimmune diseases. This positions SOCS proteins as potential therapeutic targets for modulating dysregulated immune responses in autoimmune diseases.

## 1 Introduction

Autoimmune diseases are chronic inflammatory conditions marked by abnormal local or systemic immune reactions. The body’s reactive T-cells and B-cells become overactive, triggering excessive autoantibody production and causing serious harm to multiple organs and systems (1). Their pathogenesis involves multifaceted dysregulation, with immune signaling imbalances playing a central role. Among key regulators, the SOCS family proteins function as essential checkpoints in cytokine receptor signaling pathways. These proteins are induced by various stimuli, including cytokines such as IL-6 and IFN-γ, growth factors, and hormones. By primarily targeting the JAK-STAT pathway, SOCS proteins effectively suppress hyperactive immune responses, thus ensuring the maintenance of homeostasis (2). However, unchecked cytokine signaling can propagate destructive inflammation, necessitating robust negative regulatory mechanisms. The SOCS family emerges as a critical modulator of this balance. SOCS proteins are rapidly induced by cytokines, growth factors, and hormonal stimuli, forming an essential feedback loop to restrain excessive signaling. By directly targeting key components of the JAK-STAT pathway—including receptor complexes, kinases, and transcription factors—SOCS members such as SOCS1, SOCS3, and SOCS5 dynamically calibrate immune responses to maintain homeostasis. While SOCS proteins are primarily known for regulating the JAK-STAT pathway, their influence extends beyond this. They also interact with other signaling pathways, including TAM-TLRs, NF-κB and PI3K/AKT to modulate immune responses. This broader regulatory role enhances our understanding of their therapeutic potential in various diseases.

In recent years, an increasing number of studies have shown that SOCS proteins play a significant role in the progression of autoimmune diseases such as multiple sclerosis (3),type 1 diabetes (4),and rheumatoid arthritis (5–8). Understanding these molecular mechanisms is essential to uncover the pathogenesis of these diseases and to develop new therapeutic strategies.

This review synthesizes current knowledge on SOCS protein structure, their regulatory roles in JAK/STAT signaling, and disease-specific mechanisms across major autoimmune conditions. We further evaluate emerging therapeutic strategies targeting SOCS proteins, aiming to bridge mechanistic insights with clinical translation.

## 2 SOCS

**Figure 1:**
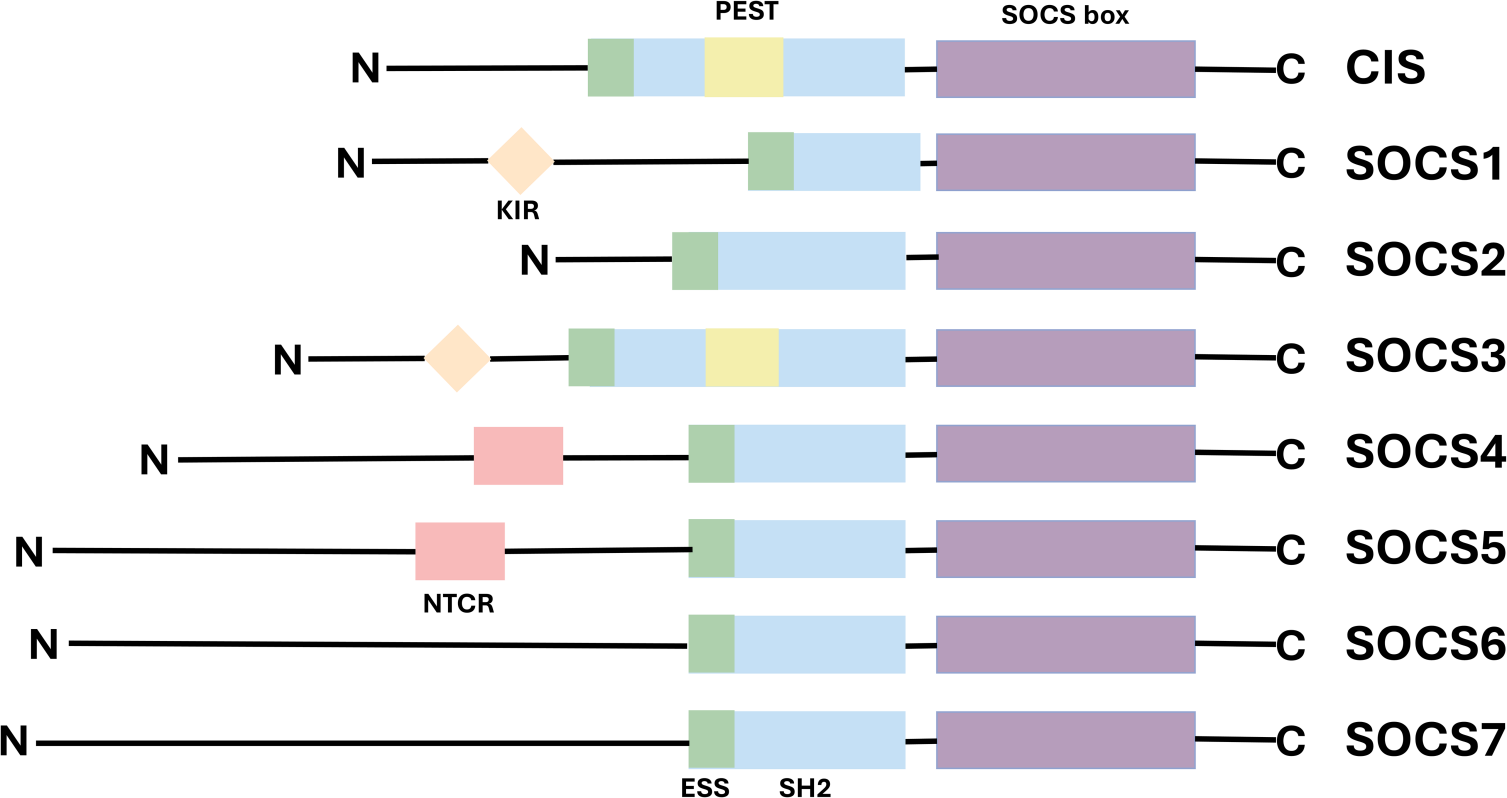
Structure of SOCS domain.

Suppressor of cytokine signaling (SOCS) represents a family of cytokine-inducible signal regulatory factors, comprising eight known member proteins: Cytokine-Inducible SH2-Containing Protein (CIS) and SOCS1-7. These proteins play a critical role in the regulation of the immune system. Their expression can be induced by various cytokines, growth factors, and hormones (9, 10). The first member of the SOCS, CIS1, was cloned as an apparently early gene induced by the conserved intracellular region of various cytokine receptors, in response to a number of different cytokines (11). The second member of the family, SOCS1, was independently cloned by three researchgroups a few years later (12–14) The SOCS proteins function as intracellular negative regulators, identified by Krebs and Hilton in the cDNA library derived from a monocytic leukemia cell line. They typically exert their effects upon activation by specific cytokines, thereby inhibiting the signal transduction process (15). Each protein contains a central Src homology 2 domain (SH2) domain flanked by variable N-terminal regions and a C-terminal SOCS box (16). SOCS proteins attach to phosphorylated tyrosine residues on JAK and/or cytokine receptor subunits via their central SH2 domain. Subsequently, the C-terminal SOCS domain interacts with the components of the ubiquitin ligation machinery, mediating the proteasomal degradation of related proteins and playing a pivotal role in inhibiting signal transduction (17). While members of the SOCS family exhibit common structural and functional conservation, each also possesses unique characteristics that manifest as structural variations at their N termini (18). For instance, SOCS1 and SOCS3 are analogous and possess homology. The N-terminal region of both has a distinctive kinase inhibitory region (KIR) that is capable of binding and inhibiting JAKs (19, 20), conversely, the N-terminal structure of SOCS2 plays a critical role in protein stability. Specifically, it can interact with JAK kinase via its SH2 domain, thereby suppressing the activation of the JAK/STAT signaling pathway. This regulatory mechanism contributes to maintaining signaling pathway homeostasis and prevents abnormal cell proliferation and differentiation that may result from excessive signal activation (21, 22). Additionally, although both SOCS4 and SOCS5 contain a conserved N-terminal region at their N termini (NTCRs), their precise functions remain to be elucidated (23) (**Figure 1**).

The SOCS protein family has 8 members, each with a C-terminal SOCS box domain (purple), a SH2 domain (blue), and an N-terminal region. The highly conserved SOCS box domain is located at the carboxy terminal. The SH2 domain is located in the center and shows homology among SOCS family members. In SOCS1 and SOCS3, there is a KIR upstream of the SH2 domain (orange), which can serve as a pseudo-substrate. The SH2 domain of CIS and SOCS3 contains a PEST (yellow). SOCS4 and SOCS5 have different N-terminal conserved regions (NTCR)(pink)

## 3 JAK-STAT

The JAK-STAT signaling pathway is composed of three key elements: Janus kinases (JAK), signal transducers and activators of transcription (STAT), and receptors (which bind to chemical signals) (24). Ligand activation primarily relies on the function of JAK kinases coupled to the receptor, thereby resulting in the phosphorylation of target cells. Cytokines, such as IL4 (25) and IL-6 (26) among interleukins (ILs) and interferons (IFNs), initiate the JAK/STAT signaling pathway by binding to their homologous receptors, after cytokines bind to receptors, the receptors dimerize or oligomerize, bringing the receptor-associated JAK molecules close to each other, thereby undergoing transphosphorylation and activation. Subsequently, JAK phosphorylates the tyrosine residues at the tail of the receptor, creating binding sites for the recruitment of STAT molecules (27). The phosphorylation of the receptor generates docking sites for cytosolic STAT, which is recruited to the receptor complex through its tyrosine phosphorylation-dependent SH2 domain. Activated and phosphorylated STAT (pSTAT) forms homo- or heterodimers via molecular-interfacial SH2-phosphotyrosine interactions, translocates to the nucleus, binds to DNA regulatory elements, modifies chromatin accessibility, and induces gene expression (28–30).

## 4 The mechanism of action of SOCS

**Figure 2:**
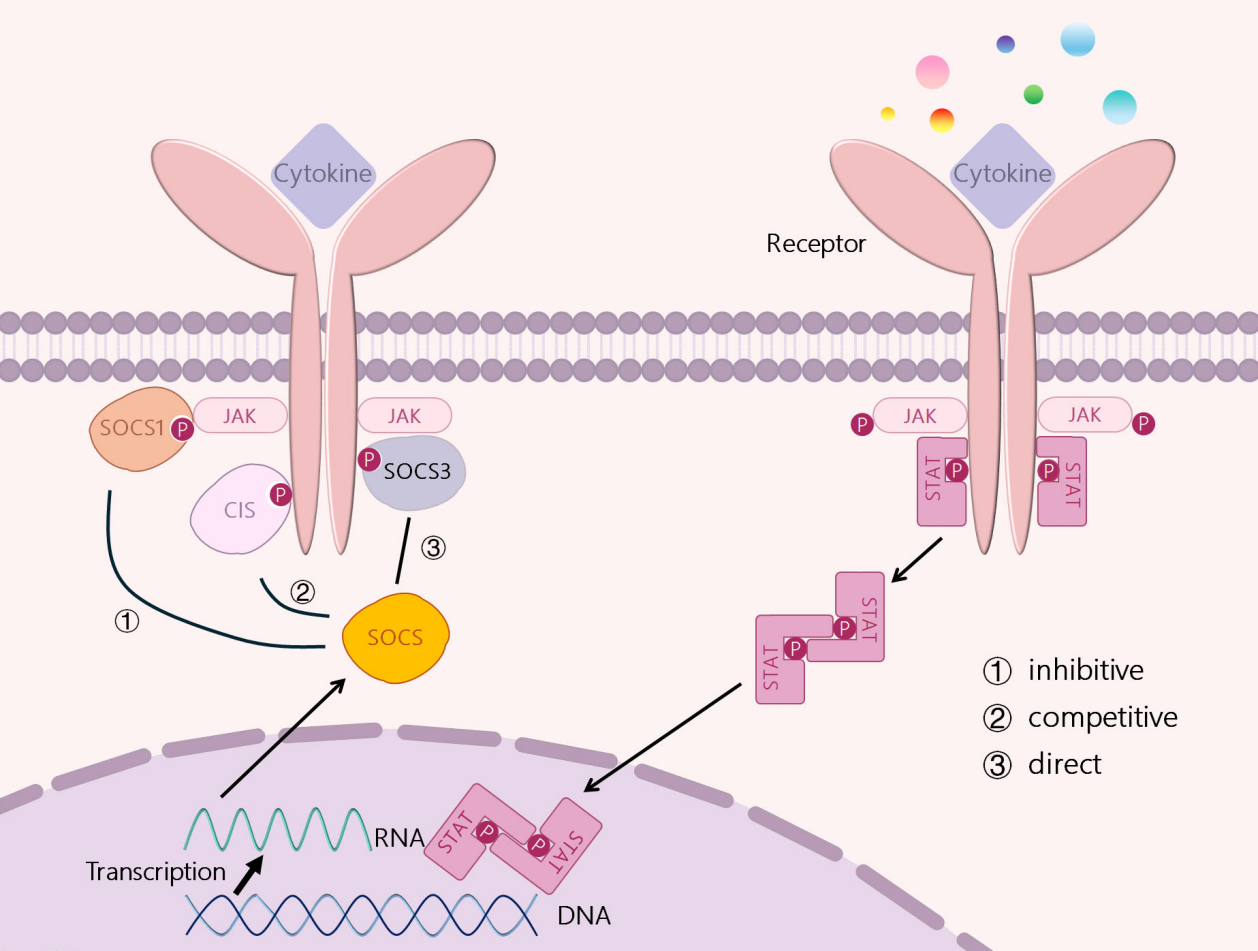
STAT-induced SOCS family proteins’ regulation of signal transduction.

SOCS can prevent the overproduction of the above mediators and down-regulate their signals, inhibiting their action (31). It has been shown that SOCS proteins can regulate cytokine receptor signaling through several different mechanisms, including inhibition of kinase activity, competitive binding, and protein degradation or rerouting, which are slightly dependent on different combinations of specific protein structural domains and motifs. (**Figure 2**) ① Inhibition of kinase activity: as mentioned above the mechanism of action of SOCS1 and SOCS3, i.e., the unique KIR at their N-terminus binds and inhibits JAKs (19, 20). ②Competitive Binding: SOCS proteins interact with specific motifs containing phosphotyrosine through their SH2 domain. thereby being able to bind to specific components of the cytokine receptor signaling complex or downstream signaling proteins (32). Competitive binding prevents STAT from binding to other proteins, thereby blocking signaling (27). ③SOCS proteins interact with phosphorylated JAK proteins or receptors through their SH2 domains, thereby promoting the formation of E3 ubiquitin ligase complexes. This process involves the addition of ubiquitin to target substrates, leading to their degradation. The SOCS-box domain of SOCS proteins plays a crucial role in this process. The SOCS-box domain can interact with the elongin B/C to form E3 ubiquitin ligase complexes, thereby promoting the ubiquitination and degradation of target proteins. This mechanism not only inhibits the excessive activation of the JAK-STAT signaling pathway but also maintains the balance of intracellular signal transduction by degrading related proteins (33). and may redirect related proteins, ultimately leading to the degradation of target proteins (34). The proteasome degradation mediated by SOCS proteins plays a critical role in regulating immune responses, yet its influence on immune homeostasis is multifaceted and complex. This degradation process may also introduce potential side effects under certain conditions. For example, SOCS1 and SOCS3 inhibit type I interferon production in human plasmacytoid dendritic cells mediated by TLR7 through the targeted degradation of IRF7. While this mechanism helps prevent excessive autoimmune activation, it may compromise antiviral immunity, as type I interferon production is essential for viral clearance (35). Consequently, SOCS-mediated degradation could potentially impair the immune system’s ability to defend against pathogens in specific contexts. In addition, SOCS2 modulates its intracellular concentration and duration by regulating its own stability, thereby dynamically controlling its inhibitory effect on signaling pathways. While this regulatory mechanism contributes to maintaining the delicate balance of signal transduction, abnormal regulation of SOCS2 expression or stability may result in dysregulation of immune responses (36). Thus, proteasome degradation mediated by SOCS proteins is vital for sustaining immune equilibrium but may also modulate the intensity and duration of immune responses. Future research should focus on elucidating the precise mechanisms of SOCS protein action in diverse immune cell types and physiological settings, as well as exploring strategies to optimize immune therapies by modulating SOCS protein activity.

CIS, SOCS1, SOCS2 and SOCS3 are all induced by STATs. CIS inhibits STAT5 activation by binding to receptors, SOCS1 can bind directly to JAKs, and SOCS3 can bind to GP130-related receptors and JAKs. SOCS2 can also bind to receptors, but it has a high specificity for GH receptors. Three mechanisms exert their signal inhibition function: ① inhibition of JAK kinase activation, ② competition for tyrosine residues, and ③ direct degradation of signal receptors.

## 5 Autoimmune diseases

Autoimmune diseases represent a category of chronic inflammatory disorders characterized by aberrant local or systemic immune responses, specifically the over-activation of the body’s own reactive T-cells and B-cells, leading to the production of an excessive quantity of autoantibodies and resulting in significant damage to multiple organs and systems. These conditions exhibit a higher prevalence among women (1). Common examples include rheumatoid arthritis, Sjögren’s syndrome, and systemic lupus erythematosus within musculoskeletal disorders; Crohn’s disease and inflammatory bowel disease among gastrointestinal disorders; Graves’ disease and Hashimoto’s thyroiditis in endocrine pathologies; as well as dermatological conditions such as psoriasis and dermatomyositis. Additionally, neurological manifestations like Guillain-Barré syndrome and multiple sclerosis are also noted. The following sections will present several typical autoimmune diseases to elucidate recent advancements in research concerning SOCS proteins in these conditions (**Table 1**).

**Table 1:** The effects of autoimmune disease-related signaling molecules.

Disease	Upstream signal	Relative SCOS	Effect	Result	Reference
MS		SOCS1↑	STAT1↓	Reduce CD40 at the protein and messenger RNA levels and slow down the progression of MS	(43, 44)
		SOCS3↓	STAT3↑	Relapse in MS	(47–49)
T1D		SOCS1↑	IL-15↓	Limit the proliferation of diabetogenic CD8 + T cells	(60)
		SOCS3↓	Higher leptin sensitivity		(35, 41)
SLE	IL-10↑	CIS↑	Th2↑	Promote disease	(69)
		SOCS1↓	IFN-γ↓ STAT1↑	Promote disease	(70–72)
	miR-155↑	SOCS1↓	Affect the stability and function of Tregs	Promote disease	(73, 74)
		SOCS3↑	IL-6↓	Exert a protective effect on the progression of glomerulonephritis and suppress the generation of autoantibodies	(75)
	miR-448↑	SOCS5↓	Th17↑	Promote disease	(76)
RA	TNF↑	CIS↓ SOCS2↓		Undetermined	(69, 85)
	miR-155↑	SOCS1↓	ERK↑	Promote disease	(6)
GD	METTL3	m6A of SOCS↑		Participate in the pathogenesis	(91)
SS’		SOCS3↑ SOCS1↑		Undetermined	(98, 99)
Uveitis		SOCS1↓	JAK2↑ IL-10↓	Intensify inflammation	(111, 112)
BD		SOCS3↑ SOCS1↑		Promote disease	(119)

## 6 SOCS and autoimmune diseases

### 6.1 Multiple sclerosis and SOCS

Multiple sclerosis (MS), also referred to as disseminated sclerosis or encephalomyelitis dissecans, is a pathological condition characterized by inflammation and immune system dysregulation (37, 38). In this disorder, the myelin sheath—the protective outer layer of nerve cells in the brain and spinal cord—sustains damage, disrupting communication within the nervous system. This disruption manifests as a diverse array of signs and symptoms encompassing physical, cognitive, and occasionally psychological dimensions (39). The pathogenesis of MS involves genetic, environmental, and immunological factors. Genetic predisposition, particularly HLA genes such as HLA-DR2+, plays a significant role (40). Environmental factors including infections, smoking, and vitamin D deficiency also contribute to the risk of MS (41, 42). Immunologically, MS is characterized by abnormal activation of T helper cells (Th1 and Th17), which release pro-inflammatory cytokines like IFN-γ and IL-17, leading to neuroinflammation and demyelination (43, 44). Correlative studies have demonstrated that overexpression of SOCS1 in murine macrophages results in decreased CD40 levels at both protein and messenger RNA levels through inhibition of STAT1 signaling induced by IFN-γ (45). Frexalimab, a monoclonal antibody targeting CD40L, is believed to obstruct the co-stimulatory CD40/CD40L pathway essential for the activation and function of adaptive (T and B cells) as well as innate (macrophages and dendritic cells) immune cells. Notably, frexalimab has been shown to attenuate new brain lesions associated with multiple sclerosis (46). Consequently, it has been hypothesized that SOCS1 may mitigate MS progression by downregulating CD40 expression. Furthermore, monocytes along with CD4+ and CD8+ T cells from patients experiencing relapsing-remitting (RR) MS exhibit reduced expression of SOCS3 during relapse compared to those from patients with remitting MS (47). During the relapsing phase of MS, elevated STAT3 activation levels may correlate with diminished SOCS3 expression—which typically inhibits STAT3 activation—potentially leading to hyperactivation of STAT3 associated with disease relapses (48). These findings provide critical insights into research related to MS treatment; however, further investigations are warranted to elucidate the specific relationship between SOCS1 and SOCS3 in relation to MS pathophysiology. Collectively, these data suggest that SOCS3 could represent an important therapeutic target for managing multiple sclerosis. The well-documented connection between decreased SOCS3 levels and heightened STAT3 activity in multiple sclerosis (MS) highlights a critical interaction driving neuroinflammation (49). In this context, reduced SOCS3 expression leads to excessive STAT3 activation, exacerbating inflammatory responses. This mechanism may also play a role in other neuroinflammatory diseases. For instance, in Parkinson’s disease (PD), diminished SOCS3 levels could contribute to STAT3 overactivation, potentially worsening microglial activation and neurodegeneration (50). Similarly, in Alzheimer’s disease (AD), decreased SOCS3 might remove an essential regulatory constraint on STAT3, thereby increasing glial cell-mediated inflammation and accelerating disease progression (51).Future research should explore whether the SOCS3-STAT3 pathway represents a common inflammatory mechanism across these conditions, as identifying such a mechanism could provide a broad therapeutic target for intervention (52).

### 6.2 Type 1 diabetes and SOCS

Type 1 diabetes mellitus (T1D) is a prevalent genetically predisposed disorder characterized by dysregulation of glucose metabolism and immune-mediated endocrine dysfunction, primarily resulting from insufficient insulin secretion due to autoimmune destruction of pancreatic β-cells. Insulin and leptin, twAKo pivotal hormones, collaboratively regulate energy balance and maintain glucose homeostasis, with their interplay further modulated by inflammatory pathways. The core immunological pathogenesis of T1D involves the autoimmune destruction of insulin-producing pancreatic β-cells mediated primarily by T-cells (53). Genetic factors significantly contribute to this susceptibility, with specific HLA alleles, such as DR3-DQ2 and DR4-DQ8, being strongly associated with an increased risk of developing T1D (54–56). Viral infections, including enteroviruses like Coxsackievirus B4, can trigger or exacerbate the autoimmune response against β-cells (57–59). Regulatory T-cells (Tregs) function abnormally in T1D patients, reducing immune tolerance and exacerbating the autoimmune attack (60). Chong et al. demonstrated that overexpression of SOCS1 in TCR transgenic NOD mice—a model for T1D—effectively prevented CD8+ T cell-mediated onset of the disease. Concurrently, elevated levels of SOCS1 were found to inhibit IL-15 expression, thereby limiting the proliferation of diabetogenic CD8+ T cells (61), indicating a protective role for SOCS1 in T1D pathology. Furthermore, studies have shown that excessive energy intake and body fat deposition caused by HFD (high fat diet) lead to resistance to leptin and insulin, whereas loss of Socs3 expression in the brain improves diet-induced obesity, leptin resistance and even insulin resistance.This suggests that the Stat3-Socs3 system in the brain plays a major role in regulating body fat mass. This study also makes brain Socs3 a potential therapeutic target for treating leptin resistance, type 2 diabetes and obesity (62).

### 6.3 Systemic lupus erythematosus and SOCS

Systemic lupus erythematosus (SLE) is a life-threatening autoimmune disease that typically results in damage to multiple organs and systems (63). It is characterized by T-cell hyperreactivity and a loss of immune tolerance to self-antigens (64). SLE arises from a combination of immune dysregulation, genetic predisposition, and environmental triggers. Key pathological mechanisms include defective clearance of nucleic acids (65), elevated type I interferon production (66), loss of B cell tolerance resulting in autoantibody formation and immune complex deposition (67), as well as the presence of genetic susceptibility loci (68). Viral infections, such as Epstein-Barr virus (EBV), may act as triggers in genetically predisposed individuals. SOCS1 plays a crucial role in maintaining immune cell homeostasis and regulating inflammation through the intricate modulation of cytokine signaling pathways. Tsao et al. demonstrated that transcript levels of CIS were significantly elevated in SLE patients exhibiting active disease compared to both healthy controls and SLE patients with inactive disease, suggesting that CIS may serve as a potential biomarker for SLE and contribute to its pathogenesis (69). Furthermore, SOCS1 may also be implicated in the development of SLE (70–72); Amir Sharabi et al. investigated the activation status of the IFN-γ signaling pathway in F1 mice with systemic lupus erythematosus (New Zealand-black × New Zealand-white) and found that the absence of SOCS1 led to impaired IFN-γ signaling, thereby exacerbating disease progression. Additionally, they observed reduced levels of SOCS1 alongside increased pSTAT1 expression in splenic-derived cells from SLE mice when compared to control groups. Subsequent experiments involving hCDR1 treatment revealed that hCDR1 could reverse these expression patterns, resulting in an improvement in clinical symptoms associated with SLE. These findings indicate diminished presence of SOCS1 in murine models affected by systemic lupus erythematosus while highlighting how hCDR1 treatment can upregulate its expression, thus restoring regulation over the IFN-γ signaling pathway (73). In the pathological progression of SLE, elevated miR-155 levels contribute to decreased SOCS1 expression, subsequently affecting Treg stability and functionality. Therefore, targeting miR-155’s regulatory mechanisms may represent a promising therapeutic strategy for ameliorating SLE symptoms (74). Potential therapeutic strategies targeting the miR-155/SOCS1 axis in SLE could include anti-miR-155 therapy to inhibit miR-155 function, thereby reducing inflammation. Alternatively, enhancing SOCS1 expression or activity, through gene therapy or small molecule activators, might help mitigate the excessive immune response. Masashi Fukuta et al. discovered that SOCS3 expression within capsule cells might exert protective effects against glomerulonephritis development by suppressing IL-6 production within these cells while inhibiting autoantibody generation through studies conducted on imiquimod-induced lupus mice models (75). This suggests that SOCS3 could potentially mitigate kidney damage arising from complications related to systemic lupus erythematosus by modulating immune responses and inflammatory processes. Given its pivotal role in regulating immune responses and inflammation, SOCS3 holds significant therapeutic implications for treating SLE. Zhang et al., However, it is noteworthy that conflicting studies have reported divergent roles of SOCS3 in lupus nephritis. Some research suggests that SOCS3 may exert protective effects by inhibiting pro-inflammatory cytokines and dampening B cell activation. Conversely, other studies propose that SOCS3 might promote disease progression under specific conditions. For example, elevated SOCS3 levels in certain immune cells could potentially exacerbate immune dysregulation. These inconsistencies underscore the complexity of SOCS3’s role in lupus nephritis and highlight the necessity for further investigation to elucidate its dual effects across different disease contexts and cell types. For the first time, in the experiments on MRL/lpr mice, inhibition of miR-448 can prevent the activation of Th17 cells and reduce the expression of IL-17A by targeting SOCS5, thereby alleviating renal injury. This indicates the association between the expression level of SOCS5 and the disease progression of SLE (76).

### 6.4 Rheumatoid arthritis and SOCS

Rheumatoid arthritis (RA) is a systemic autoimmune disease characterized by a chronic inflammatory process that can result in damage to the joints as well as various extra-articular organs, including the heart, kidneys, lungs, digestive system, eyes, skin, and nervous system (5, 7). The pathogenesis of autoimmune diseases involves complex molecular mechanisms. In rheumatoid arthritis (RA), key molecular mechanisms include the activation of proinflammatory cytokines such as TNF-α and IL-6, which drive inflammation and joint damage (77, 78). The JAK-STAT pathway is also crucial, with JAK2 playing a significant role in RA synovial tissue (78). Additionally, epigenetic alterations like DNA methylation and histone modification contribute to RA development, affecting gene expression in immune cells and synoviocytes (79, 80). Metabolic disorders, including glycometabolism and lipid metabolism, further influence the disease process by modulating immune cell functions and promoting inflammation (81, 82). These molecular mechanisms collectively lead to the chronic inflammation and joint destruction characteristic of RA. Tsao et al. experimentally demonstrated that transcript levels of CIS/SOCS genes in patients with rheumatoid arthritis were not significantly different from those observed in healthy controls; however, they noted an increase in CIS transcript expression alongside a downregulation of SOCS2 transcript levels in peripheral blood mononuclear cells (PBMCs) from RA patients treated with TNF-α blockers (69). Relevant evidence indicates that TNF may activate NF-κB (83) or indirectly activate STAT proteins through secondary cytokines (such as IL-6) (84), thereby inducing CIS expression and forming a negative feedback inhibition of the JAK-STAT pathway (69). Regarding the regulation of SOCS2, TNF upregulates SOCS2 expression by activating STAT5b (such as through the GH/IGF-1 signal cross-talk) or acting in synergy with NF-κB (83), thereby inhibiting STAT5b-mediated growth signals (85). In addition, TNF-induced oxidative stress may indirectly enhance SOCS2 activity through the Nrf2-STAT6 pathway (86). Isomäki et al. reported significantly elevated levels of SOCS1 and SOCS3 in PBMCs derived from RA patients (8). Furthermore, some scholars have proposed that LINC-PINT regulates the expression of SOCS1 by modulating miR-155-5p, thereby inhibiting the activation of the ERK signaling pathway. This mechanism suppresses both the inflammatory response and cell proliferation and invasion associated with RA pathology, highlighting the pivotal role of SOCS1 in RA pathogenesis (6).

### 6.5 Graves’ disease and SOCS

Graves’ disease (GD) is the predominant clinical subtype of autoimmune thyroid disease, representing a significant public health concern due to its substantial impact on patients’ quality of life and its association with increased mortality risk. Approximately 90% of all hyperthyroidism cases are attributable to this condition (87, 88). The pathogenesis of GD involves a Th1 immune response and elevated Th1 chemokines (89). Genetic factors and environmental triggers also contribute to GD development (90). Song et al. propose that METTL3 (Methyltransferase-like 3) may play a role in the pathogenesis of GD by inducing mRNA N6-methyladenosine (m6A) methylation modifications in members of the SOCS family (91). Given the current limitations in research within this domain, we urge more scholars to dedicate their efforts toward further validation and comprehensive studies.

### 6.6 Sjogren’s syndrome and SOCS

Primary Sjögren’s syndrome (pSS) is a common autoimmune disease that predominantly affects women (92). The hallmark of this condition is the inflammation and tissue damage of the salivary and lacrimal glands, leading to symptoms of dry mouth and dry eyes, collectively referred to as xerostomia (93). The pathogenesis of Sjogren’s syndrome (SS) involves several key mechanisms. Type I interferons (IFN) are crucial, promoting immune cell activity and contributing to salivary gland lesions (94). Th17 cells and their associated cytokines (IL-17, IL-23) play a significant role in inflammation and tissue damage (95). B cells and BAFF (B cell-activating factor) are central to the disease process, with BAFF supporting B cell survival and autoantibody production (96). Environmental factors, such as viral infections and oxidative stress, may contribute to disease initiation and progression (94, 97). These factors collectively lead to the characteristic dryness and systemic manifestations of SS. Stimuli such as IFN-α, IFN-γ, and IL-6 significantly enhance the phosphorylation levels of STAT1. Studies have observed an increased mRNA expression of suppressor factors SOCS1 and SOCS3 in patients with primary Sjögren’s syndrome (pSS). This suggests that immune cells in pSS patients exhibit a heightened response to the activation of the STAT1 signaling pathway. Such enhanced sensitivity may be one of the key factors contributing to the prominent interferon characteristics observed in pSS patients (98). In individuals with primary Sjögren’s syndrome (pSS), abnormal activation of the JAK-STAT signaling pathway leads to significant inhibitory responses. However, researchers have yet to clarify whether this phenomenon results from a deficiency in SOCS proteins, excessive activation of the JAK-STAT pathway, or a combination of both factors contributing to the pathogenesis of pSS. Multiple studies indicate that IL-6 upregulates pSTAT3 through the JAK-STAT3 pathway; furthermore, SOCS3 expression induced by pSTAT3 exerts negative feedback on JAK activity and subsequent production of pSTAT3. Despite increased expression levels of SOCS3 in pSS patients, its functionality appears diminished—potentially related to co-expression with IL-17 (99–101).

### 6.7 Uveitis and SOCS

Uveitis is a leading cause of ocular disease, accounting for 5-10% of visual impairment worldwide (102) and ranking as the fourth leading cause of blindness globally (103). Uveitis is typically induced by an eye infection, but it might also be associated with underlying autoimmune disorders. The pathogenesis of uveitis involves defects in central tolerance, allowing autoreactive T cells to escape thymic deletion and cause ocular inflammation (104). Th17 cells play a significant role by producing granzyme B, which disrupts blood-retina barriers and initiates inflammation (105). Specific HLA alleles like HLA-DR4 and HLA-B27 are associated with increased susceptibility (106). IL-23 drives Th17 responses, exacerbating disease progression (107). Dysregulation of the JAK/STAT pathway, particularly STAT3, promotes Th17 cell differentiation and inflammation (108). The analysis of cytokine expression within the eyes of uveitis patients reveals that inflammatory cytokines (such as IFN-γ and IL-17) are augmented, while the level of Tregs is decreased (109). Through the integration of human studies, equine models, and murine models of uveitis, we have identified that Th17 cells and the JAK-STAT signaling pathway play pivotal roles in the pathogenesis of this condition, with SOCS molecules being critically involved (110, 111). He et al. demonstrated that SOCS1 plays a crucial role in regulating JAK2 activity, which is essential for preventing inflammation in equine recurrent uveitis (ERU) and other autoimmune disorders. By inhibiting JAK2’s kinase activity, SOCS1 reduces inflammatory cell infiltration and pro-inflammatory cytokine expression while promoting IL-10-producing regulatory T cells. This dual mechanism not only suppresses ocular inflammation but also mitigates retinal pathology. Deficiency in SOCS1 can lead to JAK2 hyperactivation, exacerbating inflammatory responses, whereas enhancing SOCS1 function could mitigate disease progression, highlighting its therapeutic potential in uveitis (112). These findings suggest that enhancing SOCS1 function could mitigate the progression of ERU.

### 6.8 Behcet’s disease and SOCS

Behcet’s disease is a systemic vasculitis characterized by recurrent oral and genital ulcers, skin lesions, and uveitis. The condition was first identified in 1937 by the renowned Turkish dermatologist Hulusi Behçet. Research has demonstrated that this multisystem disorder can impact various organs, including the eyes, skin, joints, gastrointestinal tract, and central nervous system. Recurrent painful oral aphthous ulcers are typically the initial symptom of the disease. The pathogenesis of Behçet’s syndrome (BS) involves genetic, environmental, and immunological factors. Genetic factors like HLA-B51 and non-HLA genes (IL10, IL23R) contribute significantly (113, 114). Environmental factors such as infections and microbiome changes also play a role (115). Immunologically, hyperactivated neutrophils, increased NETosis, a Th1/Th17 cytokine profile, and upregulation of the JAK/STAT pathway are key (116, 117). Previous studies suggest a potential association between Behçet’s disease and autoimmunity (118). Hamedi et al. reported elevated expression levels of both SOCS1 and SOCS3 in patients with Behçet’s disease compared to healthy controls (HC) and those with recurrent aphthous stomatitis (RAS). Furthermore, significant upregulation of SOCS1 and SOCS3 was observed in buccal mucosal brush biopsy (BMBB) samples, revealing distinct differences between ulcerated and non-ulcerated mucosal sites (119); their increased expression may correlate with disease severity. The role of SOCS1 methylation in BD is significant. Abdi et al. investigated SOCS1 gene methylation alongside gene expression analysis and found that methylation levels of the SOCS1 gene were significantly higher in patients while its expression was reduced. This finding suggests that DNA methylation may influence SOCS1 expression contributing to the pathogenesis of Behçet’s disease. Increased methylation of SOCS1 in BD patients leads to reduced gene expression, potentially impairing its regulatory function on the JAK/STAT pathway and contributing to disease pathogenesis by exacerbating inflammation. Further mechanistic insights are needed to fully understand this epigenetic modification’s impact on BD progression (120). Based on these research findings, it is proposed that DNA methylation alterations within the SOCS1 gene could play a role in modulating its expression thereby facilitating the development of Behcet’s disease.

### 6.9 Germline SOCS1 deficiency and autoimmune diseases

SOCS1, a key negative regulator of the JAK-STAT signaling pathway, maintains immune homeostasis by inhibiting JAK kinase activity and mediating ubiquitin-dependent degradation of signaling molecules (121). Monogenic germline deficiency of SOCS1 (such as heterozygous frameshift or missense mutations) leads to loss of its function, triggering broad autoimmune and autoinflammatory phenotypes (121). Studies have shown that SOCS1 haploinsufficiency enhances immune cell sensitivity to cytokines (e.g., IFN-γ, IL-2, IL-4), manifested by elevated STAT1/5/6 phosphorylation and abnormal expression of downstream genes (e.g., CXCL9, CISH). This hyperactive signaling drives T cell hyperproliferation and regulatory T cell (Treg) dysfunction, disrupting immune tolerance (121, 122). As SOCS1 functions as an E3 ligase substrate it is also involved in FAK-AKT-RS6K and TLR signaling (122). Körholz J et al. demonstrated that SOCS1 haploinsufficient patients cells showed an increased activity in the FAK– AKT– RS6K pathway further explaining the accumulation of autoimmunity. And in the study of Körholz J et al., it was observed that the cytokine responses of SOCS1 haploinsufficient patients to multiple TLR ligands (TLR2–5 and TLR7-9) were enhanced. Clinical investigations show that SOCS1-deficient patients commonly develop early-onset autoimmune diseases, including Hyper IgE-like syndrome (HIES) with eczema and purulent infections, systemic lupus erythematosus (SLE) with cutaneous and renal involvement, autoimmune cytopenias (e.g.,immune thrombocytopenia, Evans syndrome), and organ-specific autoimmunity (e.g., psoriasis, thyroiditis) (121, 123). Autoinflammatory phenotypes include chronic recurrent multifocal osteomyelitis (CRMO), juvenile idiopathic arthritis, and intestinal inflammation, which may result from enhanced monocyte responses to TLR ligands and increased secretion of proinflammatory factors (e.g., IL-8, IL-18) (122, 123). Notably, the serum cytokine profile in these patients resembles that in STAT1/3 gain-of-function mutations, indicating that hyperactivation of the JAK-STAT pathway is a shared pathogenic mechanism (121). Additionally, SOCS1 deficiency amplifies immune inflammation by dysregulating the FAK1-AKT-RPS6K pathway and TLR signaling (122). Targeted therapy studies show that JAK1/2 inhibitors (e.g., ruxolitinib) suppress excessive STAT phosphorylation in patient cells (121), while IL4Rα and IL17A blockers exhibit marked efficacy in patients with comorbid atopic dermatitis and arthritis (124). These findings highlight the clinical potential of precision interventions targeting hyperactive signaling pathways.

The above collected studies of SOCS in several typical autoimmune diseases in recent years, which revealed an important relationship between the SOCS family and a variety of typical autoimmune diseases.

## 7 SOCS in the treatment of autoimmune diseases

Through the analysis of the role of SOCS (**Table 2**) and the association between SOCS and various diseases, researchers have progressively elucidated the pivotal role of SOCS in regulating immune responses and maintaining immune homeostasis. These findings offer novel insights and potential targets for future clinical interventions. For instance, strategies aimed at modulating SOCS expression may facilitate the restoration of immune equilibrium and mitigate autoimmune responses, thereby enhancing patient prognosis. Consequently, these studies not only provide a theoretical framework for understanding disease pathogenesis but also establish a foundation for developing innovative therapeutic strategies, particularly immunomodulatory therapies that target the SOCS pathway. Sukka-Ganesh B. and Larkin J. III suggest that therapeutic approaches focusing on SOCS1 signaling could be effective in treating SLE (70). Palmroth M., Kuuliala K., et al. demonstrated that tofacitinib reduces SOCS3 expression by inhibiting multiple JAK-STAT pathways in patients with rheumatoid arthritis (125). Li XL, Zhang B., et al. reported that rapamycin administration regulates innate immunity primarily through modulation of the TAM-TLRs-SOCS signaling pathway while decreasing levels of both SOCS1 and SOCS3; this intervention alleviates neurological deficits and myelin sheath loss in experimental autoimmune encephalomyelitis (EAE) mice, providing a robust theoretical basis for multiple sclerosis treatment (126). Enhancement of the expression of the SOCS1 gene can suppress the signal transduction mediated by IFN-γ-activated STAT1, thereby reducing the quantity of CD4+ T cells related to multiple sclerosis. The experimental outcomes demonstrate that the analogue of the SOCS1 protein - a tyrosine kinase inhibitor peptide (Tkip) - is capable of interacting with the self-phosphorylated form of JAK2 to regulate the pathological process in the animal model of multiple sclerosis (127). Plummer CE., Polk T., et al. found that topical application of an SOCS1-KIR peptide relieves symptoms associated with equine recurrent uveitis (111). The local application of R9-SOCS1-KIR is also capable of inhibiting the inflammatory activity of IFN-γ, TNF-α, and IL-17, precluding the occurrence of experimental autoimmune uveitis (EAU) in mice and thereby safeguarding mice from ocular lesions (128). As research into autoimmune diseases advances, their underlying mechanisms are increasingly well characterized; concurrently, the significant role played by the SOCS protein family in disease pathogenesis is being systematically explored. Currently, considering modulation of SOCS as a therapeutic direction has gained traction to further alleviate disease progression; however, integrating SOCS with JAK-STAT signaling pathways as a treatment strategy presents challenges due to potential aberrations in both expression and function across different autoimmune states (18). The complexity and interactivity inherent within immune responses may lead to scenarios where SOCS antagonize cytokine-mediated inhibition within negative feedback regulation of the JAK/STAT pathway—resulting in inhibitory cross-talk among components involved. Moreover, genetic predisposition along with aging factors can pose risks and challenges to this regulatory pathway. Notably, there is a need to consider potential off-target effects when targeting SOCS proteins, as these may interact with other signaling pathways and disrupt normal cellular functions (129).Additionally, the therapeutic delivery of SOCS-targeted agents poses significant challenges, including ensuring specificity and efficiency of delivery to the desired cells or tissues. Furthermore, compensatory pathways may emerge in response to SOCS modulation, potentially reducing therapeutic efficacy and necessitating a more comprehensive understanding of the immune system’s adaptive mechanisms (130).

**Table 2:** Single-gene information of SOCS.

Gene	Location [Homo sapiens (human)]	Length (bp)	Function	Reference
CIS	Chromosome 3, NC_000003.12 (50606489.50611774, complement)	5,286	Competitive binding	(32)
SOCS1	Chromosome 16, NC_000016.10(11254417.11256204, complement)	1,788	JAK inhibition Degradation Ubiquitination	(19, 20) (35) (33)
SOCS2	Chromosome 12, NC_000012.12 (93569969.93626236)	56,268	JAK inhibition Ubiquitination	(36) (33)
SOCS3	Chromosome 17, NC_000017.11 (78356778.78360925, complement)	4,148	JAK inhibition Degradation	(19, 20) (35)
SOCS4	Chromosome 14, NC_000014.9 (55027236.55049489)	22,254	JAK inhibition Degradation	(9)
SOCS5	Chromosome 2, NC_000002.12 (46698937.46763129)	64,193	Competitive binding	(32)
SOCS6	Chromosome 18, NC_000018.10 (70289045.70330199)	41,155	Ubiquitination	(33)
SOCS7	Chromosome 17, NC_000017.11 (38351844.38405593)	53,750	Ubiquitination JAK inhibition	(131)

## 8 Conclusion and prospects

In conclusion, this review explores the structure of SOCS and its regulated JAK/STAT pathway and summarizes the molecular complexity of the regulatory mechanisms by which SOCS proteins play a role in multiple sclerosis, type 1 diabetes mellitus, systemic lupus erythematosus, Graves’ disease, Sjögren’s syndrome, uveitis, rheumatoid arthritis and leukodystrophy in recent years. In addition, the article reviews the latest research advances in the treatment of autoimmune diseases in recent years, providing new therapeutic strategies and research directions for the field.

However, this review has several limitations: current research on the SOCS family and autoimmune diseases predominantly focuses on SOCS1 and SOCS3, with insufficient comprehensive discussion regarding the specific roles of each component within the molecular structure of SOCS proteins. Other family members, such as SOCS2, SOCS4-SOCS7, and CIS, remain underexplored; thus, further in-depth studies are warranted to ascertain whether these additional members contribute to the pathogenesis of various diseases and elucidate their mechanisms of action. To address the underexplored SOCS family members, research could focus on elucidating SOCS2’s role in rheumatoid arthritis (RA) through expression analysis and functional studies. Specifically, this could involve evaluating SOCS2 expression levels in RA patient samples and employing CRISPR-based techniques to manipulate SOCS2 expression in RA cellular models, thereby clarifying its impact on inflammatory responses and joint damage. The influence of CIS on autoimmune T cell responses can be explored by analyzing its expression in T cells from RA patients and assessing how its modulation affects T cell activation, proliferation, and cytokine production. For SOCS4-SOCS7, studies might systematically investigate their expression patterns, interactions with autoimmunity-related pathways, and therapeutic potential. Advanced techniques such as single-cell RNA sequencing could provide insights into the heterogeneity of SOCS protein expression across immune cell subsets, potentially uncovering novel roles for these understudied family members. These research directions aim to fill existing knowledge gaps and deepen our understanding of the SOCS family’s contributions to autoimmune pathogenesis.

Additionally, this review did not address the intricate interactions and cross-talk mechanisms between various cytokines and SOCS proteins that regulate the JAK-STAT pathway. The investigation into the TAM-TLRs-SOCS, NF-κB and PI3K/AKT signaling pathway also requires deeper exploration to enhance our understanding of its relationship with disease processes and provide a theoretical basis for diagnosis and treatment strategies. Moreover, it is essential to determine whether other autoimmune conditions—such as myasthenia gravis and Hashimoto’s thyroiditis—are associated with components of the SOCS family while conducting more extensive research into their mechanisms in newly identified SOCS-related diseases. Comprehensive treatment-related experiments must be undertaken as well. Despite promising results from therapeutic strategies modulated by SOCS in rodent models, we must acknowledge that experimental methods along with inherent limitations may not fully replicate human physiology; this discrepancy could restrict the generalizability of findings. Given the critical role played by SOCS proteins in regulating immune responses alongside their potential therapeutic applications, there is an urgent need for developing more comprehensive, safer, and effective inhibition strategies applicable across a broader range of scenarios.

In summary, exploring both the relationship between SOCS proteins and autoimmune diseases as well as their prospective clinical applications remains an active area of inquiry. To improve treatment outcomes and enhance quality of life for patients suffering from autoimmune disorders, future research should persistently investigate how these proteins function within disease contexts while striving toward innovative therapeutic approaches. Technological innovation is essential for addressing these gaps. CRISPR-based saturation mutagenesis can identify residues critical to SOCS function, while cryo-EM structural analysis of SOCS-receptor complexes (e.g., SOCS3-gp130) could provide insights into the design of isoform-specific inhibitors. Machine learning-driven modeling of cytokine networks may further elucidate SOCS-dependent signaling thresholds in autoimmune microenvironments. However, translating these advancements into clinical applications presents human-specific challenges, such as SOCS isoform diversity and off-target effects. Prioritizing organoid-based drug screening and nanoparticle-mediated delivery systems could help overcome these obstacles, thereby enhancing the clinical applicability of SOCS-targeted therapies. Such studies will illuminate not only pathogenic mechanisms underlying autoimmune diseases but also offer vital insights into novel treatment development strategies. Therefore, ongoing research coupled with technological innovation holds great promise for revolutionizing management practices related to autoimmune disorders.
